# Collision indicator charts for gantry‐couch position combinations for Varian linacs

**DOI:** 10.1120/jacmp.v12i3.3405

**Published:** 2011-03-02

**Authors:** Stewart J. Becker

**Affiliations:** ^1^ Department of Radiation Oncology New York University Langone Medical Center New York NY 10016 USA

**Keywords:** collision, treatment planning, gantry, couch, non‐coplanar

## Abstract

The use of non‐coplanar radiation fields can potentially lead to collisions between the gantry and the couch or patient. The collisions are often not realized until the plan is finished and the fields are checked on the machine, or even later when the patient is already on the table. This paper presents an easy method of gauging if a collision is likely between the gantry and couch or patient during treatment planning. The method involves creating a chart of allowable gantry and couch combinations. The charts contain curves on a polar graph of the gantry and couch angle “plane”. The curves display the limits of collisions for each gantry and couch combination for vertical couch positions 10, 15 and 20 cm below isocenter and for couch lateral positions of −10,0, +10cm, covering the majority of couch positions encountered in patient treatments. All combinations in the region within the curves (containing the origin) are valid, while all combinations outside the curves will result in a collision. The data for the charts are collected from measurements of the gantry angle that just clears each couch angle. The patient presence was modeled by placing a stereotactic body frame on the top of the couch. Separate charts were created for couch angles between 0° and 90° and between 360° and 270° over all gantry angles. The graphs are easy to create, implement, and use in the clinic and help reduce the time, complications, and uncertainties of planning with non‐coplanar fields.

PACS numbers: 87.55.‐x; 87.56.‐v

## I. INTRODUCTION

The treatment couch position may sometimes be required to deviate from its neutral position during treatment in order to improve a planned dose distribution as measured by coverage of the target and sparing of surrounding normal structures. Treatment planning for several anatomical sites requires couch rotations while the gantry is also rotated in order to achieve optimal dose distributions. The use of couch rotations allows the dose to be spread out longitudinally, reduces hot spots in the body, and often improves conformality. However, if one is not careful, the use of couch rotations may also result in couch‐gantry and patient‐gantry collisions. A few authors have reported on computer programs for virtual collision detection using geometric calculations.^(^ ^1^ ^–^ ^8^ ^)^ These programs, while useful, are difficult to create or can be awkward for routine use in an average clinic (e.g., the software is on a different computer or workspace; they require manual data inputs). The purpose of this paper is to present a method of creating a series of collision charts that can be easily printed out or reproduced and used in the planning process without the need to create, install, and use separate software. This paper will also present ready‐made charts for the Varian Clinac system. These charts cover a range of common couch heights and lateral offsets for the full range of couch and gantry angles. The charts are to be used as a guide in the planning process. The planner can quickly tell whether the gantry‐couch combination will clear or results in a collision, or whether it needs to be validated on the machine if it is close. As couch height and lateral offset determine what gantry‐couch combinations are allowable, separate charts were created for each lateral offset that includes multiple couch vertical positions. These couch positions cover the majority of treatments. The chart was created for a Varian 21EX linac with Millennium multileaf collimator (MLC) and Clinac exact couch.

## II. MATERIALS AND METHODS

Measurements were performed on a Varian 21EX – Exact Couch combination with a Millenium MLC (Varian Medical Systems, Palo Alto, CA). The convention for gantry and couch angles are: a) gantry: 0° when beam is pointing vertically down, increasing angles in the clockwise (facing the gantry) direction, and b) couch: 0° is the nonrotated, neutral position with 90° being to the left and 270° to the right, when facing the machine (clockwise increase when viewed from above). Couch positions between 90° and 180° or 180° and 270° are not reachable.

Patient plans were reviewed to determine the most common vertical couch positions and lateral offsets, and their respective ranges utilized in cases that require couch rotations such as SBRT. Couch vertical positions of 10, 15, and 20 cm were selected since they encompassed the vast majority of the cases. That is, the couch top moves below the isocenter by 10, 15 or 20 cm. Couch lateral offsets of 0 and ± 10cm were chosen as representative offsets. The positive direction is to the right when looking at the gantry (patient's left when lying supine on the table).

In order to determine the maximum couch angle that is achievable for each gantry position, the gantry was rotated at 5° intervals and the couch was moved until it was within 1° of colliding with the gantry. Measurements were taken at higher sampling rate near collision zones. Measurements were taken without the accessory tray.

The patient's presence was simulated by placing an Elekta stereotactic body frame (23 cm high and 48.5 cm wide) on the table (Elekta, Stockholm, Sweden). Any near collisions with the frame were assumed to be patient collisions also.

## III. RESULTS

The measurements for couch and gantry clearance were collected and graphed with MATLAB (The MathWorks, Natick, MA). (Figures 1a)and (1b), 2(a) and 2(b), and 3(a) and 3(b) are of couch lateral offsets of 0, +10, and −10, respectively. The first figure of each pair (1(a), 2(a), and 3(a)) are of couch angles between 0° and 90°, and the second figure of each pair (1(b), 2(b), and 3(b)) are of couch angles between 360° and 270°. Overlaid on each polar style graph are the results for the three couch vertical positions discussed, 10 cm, 15 cm or 20 cm below the isocenter. The graphs may be read by finding the gantry angle on the outside of the polar plot and then moving in and determining at which radii, couch angle, one of the curves is intercepted. The innermost intersection is the most conservative estimate of a collision‐free combination of gantry and couch angles. The couch angle is read from the radial scale. It can be seen from the figures that the most conservative estimate for safe gantry‐couch angle combination is not always associated with the same couch height. This reflects consideration for anterior versus posterior oblique gantry angles. Every combination on the “inside” of the curves (the region containing the origin) is valid and free of collisions. In addition, there are alternate renderings of the graphs that can be employed. For example, some of our planners prefer to have only one couch height per graph and have more pages. Shading the regions of the graph that are collision zones makes it easier for them to see what combination leads to a collision.

**Figure 1(a):**
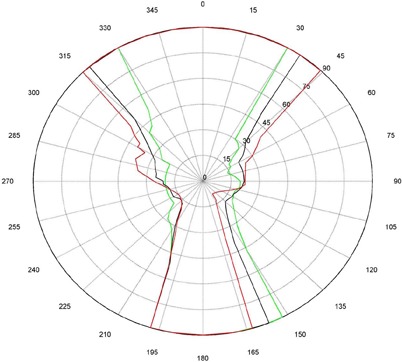
Couch vs. gantry chart for vertical couch positions of 10 cm (green), 15 cm (black), and 20 cm (red), lateral offset of 0 cm, and couch rotations from 0° to 90°. Every angle inside the lines (including the origin) is collision‐free.

**Figure 1(b):**
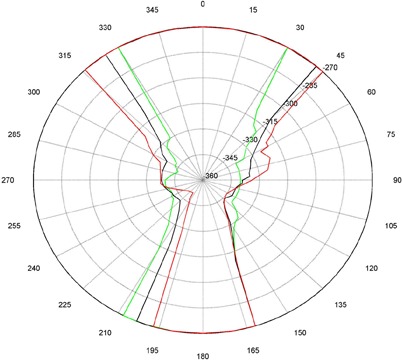
Couch vs. gantry chart for vertical couch positions of 10 cm (green), 15 cm (black), and 20 cm (red), lateral offset of 0 cm, and couch rotations from 360° to 270°. Every angle inside the lines (including the origin) is collision‐free.

**Figure 2(a):**
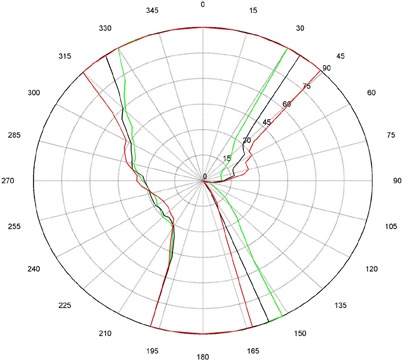
Couch vs. gantry chart for vertical couch positions of 10cm (green), 15cm (black), and 20cm (red), lateral offset of +10cm, and couch rotations from 0° to 90°. Every angle inside the lines (including the origin) is collision‐free.

**Figure 2(b):**
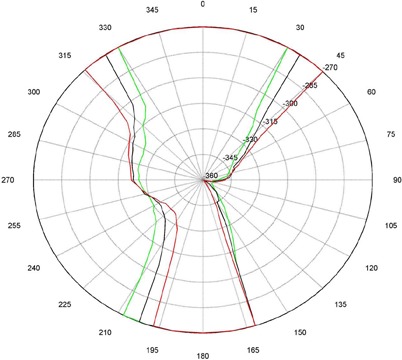
Couch vs. Gantry chart for vertical couch positions of 10 cm (green), 15 cm (black), and 20 cm (red), lateral offset of +10cm, and couch rotations from 360° to 270°. Every angle inside the lines (including the origin) is collision‐free.

**Figure 3(a):**
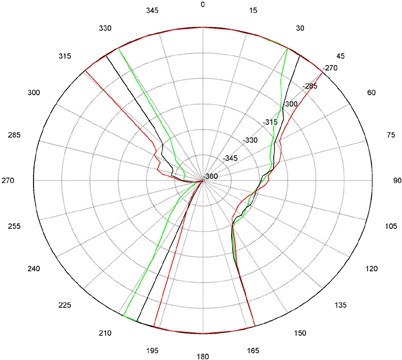
Couch vs. gantry chart for vertical couch positions of 10 cm (green), 15 cm (black), and 20 cm (red), lateral offset of −10 cm, and couch rotations from 0° to 90°. Every angle inside the lines (including the origin) is collision‐free.

**Figure 3(b):**
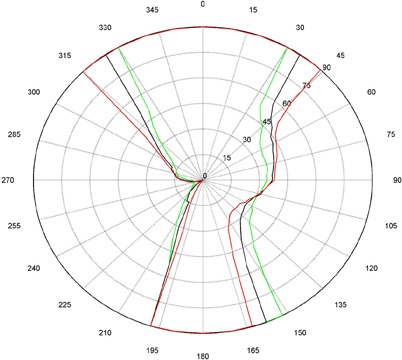
Couch vs. gantry chart for vertical couch positions of 10 cm (green), 15 cm (black), and 20 cm (red), lateral offset of −10 cm, and couch rotations from 360° to 270°. Every angle inside the lines (including the origin) is collision‐free.

## IV. DISCUSSION

There are some interesting features present in the graph. First, there are sharp “bumps” on the curves. These result from the gantry colliding with different components of the couch such as the base, the rails, and all the sharp corners under and on the side of the couch. Small gantry angle differences can cause the collision spot to change and result in a much different clearing couch angle. It should be noted that these figures are specific for Varian linacs that fit the 21EX model equipped with the Exact Couch tabletop.

The second readily‐noticeable feature is the asymmetry under the table. When the couch is rotated towards the gantry, the gantry always collides with the base of the couch no matter what vertical position is used. For instance, in Fig. 1 at approximately 165°, the couch and gantry collide for all couch heights. However, when the couch is rotated away from the gantry, the end of the couch is more easily cleared then the base of the couch. All the figures show that when the couch is raised, the clearance under the couch is greater and the clearance above the couch is less. The figures with the lateral offsets also show an asymmetry, especially around gantry angles 90° and 270°.

While this chart is easy to navigate, it does have limitations: 1) accuracy of the measurements, 2) determining lateral offset from planning CT, 3) collisions with the patient using anterior fields, and 4) variability in couch longitudinal. The accuracy of the chart is determined by the number of angles measured. However the more accurate (finer resolution) the chart, the more time it takes to perform all the measurements and create the chart. One way to deal with the large amount of measurements is to use a variable measurement increment. A smaller measurement increment can be used where the changes occur rapidly in the couch gantry combinations and larger increments where they change slower. Any combination near the curve should be checked before the plan is approved and the patient is on the table. The range of useable couch parameters will also determine how many measurements are taken. We chose three couch heights (10 cm, 15 cm, and 20 cm from isocenter) and three lateral couch offsets (neutral and ± 10cm). These positions were chosen because they cover the vast majority of couch positions used at our clinic. However, there are a few setups that might require a couch vertical position of greater than 20 cm, or lateral positions greater than 10 cm away from the central position and, in these cases, the gantry‐couch combinations need to be checked on the machine.

The lateral offset is often difficult for the planner to determine based on the CT. In this instance, all a planner can do is estimate the offset based on the distance between the isocenter position and the middle of the patient on the CT. Estimating the possible collision points with a patient when measuring anterior fields may also be problematic. The SBRT frame was used as a surrogate for the patient to simulate collision with the body. However, collisions with the arms and head of the patient are harder to estimate. Therefore, conservative angles were chosen. The patient would most likely get nervous about a collision well before there would actually be one.

The couch longitudinal position introduces another level of uncertainty. The couch table top is not uniform in its construction; therefore, its position can change the clearance values. The couch has a thick end piece, moveable bars, and hand rails. Depending on the couch longitudinal position, the point of collision can change. These measurements represent the most conservative. All of these uncertainties resulted in a graph that was more conservative (less clearance) than the “true” collision chart. Having slightly smaller angles available to use is preferable to discovering a potential collision with a patient in the treatment room.

There are multiple ways to present the data, from all curves on one graph to every curve having its own graph. We felt that when using more than three couch positions per graph and two pages of graphs, it becomes cumbersome to keep track of what is being presented. The purpose of the graphs is to supply a quick reference that can be an easy aid in planning. With too many pages or lines on the graph, the system will be too cumbersome to use. At that point, a program such as the ones referenced earlier would be easier to use. These programs use either extensive measurements or complex modeling and calculations to determine the gantry couch combinations that cause collisions. These programs can be built to cover a much larger range of couch positions but at the cost of being more difficult to use and create. These charts, unlike custom computer programs, are literally at the fingertip of the planner, and the data for the charts can be easily obtained in a few hours of work.

## V. CONCLUSIONS

This simple chart can be printed out or easily recreated for a particular linear accelerator. It allows for quick determination if a gantry‐couch combination is going to clear, or whether it may require special investigation at the machine. As a result, the use of these charts greatly reduces the number of angles that need to be verified at the machine during the planning process. Perhaps more importantly, it should reduce the number of times that a plan needs to be modified due to angles not clearing during the patient setup. Our clinical experience has shown that planners have an easier time referring to the sheet of paper than trying to manipulate another program on an already crowded computer screen.

## ACKNOWLEDGMENTS

I would like to thank Keith DeWyngaert, Gabor Jozsef, Allison McCarthy, and Christine Hitchen for their insightful input and detailed editing of this paper.
